# Address by Prof. Coventry, and Report on Dislocations, by Prof. Hamilton

**Published:** 1855-08

**Authors:** 


### EDITORIAL DEPARTMENT.

*Address by Prof. Coventry, and Report on Dislocations, by Prof. Hamilton.*—"The Philosophy of Medicine as a Science and an Art," is the title of the annual address before the Medical Society of the State of New York, by the late President, Prof. Charles B. Coventry, of Utica. It bears the impress of the practical good sense and philosophical mind of the author, a gentleman long distinguished as an able teacher and practitioner, and who has deeply at heart the dignity, honor, and usefulness of the profession. Prof. C. defines medicine, "the department of natural science which treats of the means of preventing diseases and of removing them when actually present;" and he illustrates the positions that "medicine is both a science and an art, both having the same great object, the amelioration of human suffering, but still separate and distinct; that the science of medicine, depending as it does on the fixed laws of creation, is unchangeable, whilst the art of medicine depending on the skill and tact of the physician and surgeon, must vary with each particular case; the term science means knowledge, and has been defined, knowledge reduced to principles; whilst art is defined, knowledge reduced to practice." As a department of natural science, medicine deals with facts and general laws not less real and immutable than those of the inorganic creation. The logical methods which pertain to medical investigations do not differ essentially from those suited to the prosecution of other natural sciences. The same care and caution are requisite as in the study of chemistry or natural philosophy. It must be admitted, however, that medicine is the most extensive, and, at the same time, the most obscure and difficult of all the branches of natural science. To quote the language of the author: "The chemist can retire to his laboratory, and in silence and alone test the truth of his discoveries. If he hears of discoveries made by others, he has but to submit them to the test of the crucible and blow-pipe. The natural philosopher can force nature to the test and compel her to reveal her secrets. The physician has no such power; he is compelled to wait in patience nature's own time, until the opportunity offers, and then, instead of an inanimate mass, which may be moulded and tested at pleasure, you have a living, moving body, which is constantly changing—never stationary—animated by a spirit, and where all the ordinary laws which govern matter are disregarded or removed; well might the father of the medicine exclaim, 'Ars longa, vita brevis, experientia dificile'—so difficult, indeed, that some persons of a sceptical mind have considered it unattainable."

In spite of the difficulties to be encountered, and although much time and labor have been fruitlessly expended in building and defending idle theories—"attempts to substitute the imaginations of man for the laws of the Creator"—much has been accomplished, "and at no time has the true science of medicine been cultivated with more zeal and success than at the present day."

In the application of the principles of the science to the prevention and cure of disease, and the alleviation of human suffering, consists the art, as distinguished from the science of medicine. Here great injustice is done to the medical profession by the oft repeated reproach that "doctors differ." The differences in practice are generally more apparent than real. Frequently there is entire agreement as to the particular ends to be attained by treatment, but these ends are attainable by different means, and it is chiefly in the choice of that, the latter, that practitioners are not in unison.

With reference to an impression not unfrequently entertained, that the practice of medicine involves a system of rules which, if a person be peculiarly apt, may be easily mastered, Prof. C. remarks: "Medicine is no exception to the universal rule, that the qualifications of the individual will be in proportion to his natural capacity, his opportunity for improvement, his experience and habits of observation. The mere assumption of a name, as allopath, homœopath, hydropath, or chrono-thermal, can never qualify a man for the responsible duties of the medical profession who is disqualified by nature, or who has not qualified himself by study and observation. True medicine knows no such distinctions, and the true physician is content with the designation which the name imports, viz: a student of nature."

These views, to the intelligent physician, are not new; but they are clearly and forcibly presented, and considering that the address was delivered in the legislative hall, before a mixed audience, the subject was as judiciously selected, as it was ably handled.

The address concludes with brief allusions to some of the benefits conferred on the world by medical science and art. This plain statement speaks more than volumes of illustrations: "The value of life has been nearly doubled during the last century." The author refers in terms of strong eulogium to the recent report of the sanitary commission of New Orleans, as settling the vexed question of the non-contagiousness of yellow fever, if not of cholera; and proving demonstratively that the former of these epidemics is of domestic origin, depending on a combination of causes, some of which are controlable. This doctrine, he states, was urged in an address before the New York State Society, thirty-three years ago, by its president, the father of the speaker, Dr. Alexander Coventry. Of the future advantages to the race which may be expected to flow from the continued application of medical science and art to the prevention of disease, views are adopted which may seem extravagant to those who have not given to the subject adequate consideration; but to all who are in any measure conversant with its merits, it must be conceded to take precedence of all others save those which concern directly the intellectual, moral, and religious nature of man; and with the latter it sustains relations by no means insignificant.

Prof. Coventry's address is contained in the Transactions of the New York State Medical Society, for 1855. Following the address, is an elaborate and able report on Dislocations, by Prof. F. H. Hamilton, the successor of Prof. Coventry to the presidency of the society. Prof. Hamilton's report is not of a character to admit of a full analytical notice. He presents a collection of cases, with a condensed history of each case, and the results carefully tabulated. It is with reference to these results especially that the facts laboriously gathered and collected in this paper, are of great importance. An idea may be formed of the extent of the researches, from the number of cases of the different species of dislocation which are analyzed: No. of cases of dislocation of the clavicle, 9; of the humerus, 44; of the radius, 14; of the radius and ulna, 21; of the thumb, 7; of the fingers, 5; of the femur, 6; of the tibia, 17; of the tibia and fibula, 4; of the tarsal bones, 2; of the metacarpal bones, 2. The sum total of cases analyzed is 141. These cases are not gleaned from reports by others, but have occurred under the reporter's own observation, during a surgical practice of twenty-one years, the notes having been written by his own hand at the time of the observation of each case. The aggregation of the recorded histories of these 141 cases has required a long period, and much perseverance, but the reward of well-directed industry, although in some instances it may be postponed, is sure to come. And the true reward of scientific industry is the satisfaction of knowing that it has not been fruitless. That our distinguished friend, the author of this report, has a right to enjoy this honorable recompense for many years of toil, will not be denied by the reader of his reports on dislocations, and on fractures, the latter being about to appear in the Transactions of the American Medical Association for the present year. Contributions to science of this description are not ephemeral, but will confer distinction in years to come, when others, stimulated by the examples of those who have gone before them, shall succeed the laborers of the present day. A. F.

